# What factors affect patients' recall of general practitioners' advice?

**DOI:** 10.1186/1471-2296-12-141

**Published:** 2011-12-28

**Authors:** Polona Selic, Igor Svab, Marija Repolusk, Nena K Gucek

**Affiliations:** 1 Department of Family Medicine, Faculty of Medicine, University of Ljubljana; Poljanski nasip 58, 1000 Ljubljana, Slovenia

## Abstract

**Background:**

In order for patients to adhere to advice, provided by family doctors, they must be able to recall it afterwards. However, several studies have shown that most patients do not fully understand or memorize it. The aim of this study was to determine the influence of demographic characteristics, education, amount of given advice and the time between consultations on recalled advice.

**Methods:**

A prospective survey, lasting 30 months, was conducted in an urban family practice in Slovenia. Logistic regression analysis was used to identify the risk factors for poorer recall.

**Results:**

250 patients (87.7% response rate) received at least one and up to four pieces of advice (2.4 ± 0.8). A follow-up consultation took place at 47.4 ± 35.2 days. The determinants of better recall were high school (OR 0.4, 95% CI 0.15-0.99, p = 0.049) and college education (OR 0.3, 95% CI 0.10-1.00, p = 0.050), while worse recall was determined by number of given instructions three or four (OR 26.1, 95% CI 3.15-215.24, p = 0.002; OR 56.8, 95% CI 5.91-546.12, p < 0.001, respectively) and re-test interval: 15-30 days (OR 3.3, 95% CI 1.06-10.13, p = 0.040), 31-60 days (OR 3.2, 95% CI 1.28-8.07, p = 0.013) and more than 60 days (OR 2.5, 95% CI 1.05-6.02, p = 0.038).

**Conclusions:**

Education was an important determinant factor and warrants further study. Patients should be given no more than one or two instructions in a consultation. When more is needed, the follow-up should be within the next 14 days, and would be of a greater benefit to higher educated patients.

## Background

General practitioners' (GP) work is becoming increasingly complex. The majority of patients are older, and the incidence of multi-morbidity in Slovenia is increasing [1]. In Canada, one study showed that 9 out of 10 patients had more than 1 chronic condition [2]. The prevalence of having 2 or more medical conditions in the 18- to 44-year, 45- to 64-year, and 65-year and older age-groups was, respectively, 68%, 95%, and 99% amongst women, and 72%, 89%, and 97% amongst men, with the mean number of conditions increasing significantly with age [2]. Recent data on multi-morbidity in England [3] revealed that 16% of patients had more than one chronic condition, accounting for 32% of all consultations. Using different criteria, 58% of people had multi-morbidity and they accounted for 78% of consultations [3].

Consequently, patients receive large amounts of new information and medical advice at a single consultation. In order for patients to adhere to this advice, they must be able to recall it afterwards; however, several studies have shown that most patients do not fully understand or memorize it. Lack of understanding and recollection reduces patient satisfaction and commitment to treatment [4-6]. Recall is affected by the use of medical terminology (which is difficult to understand), the form of the given information (oral or written), and patients' expectations and level of education [6,7].

Recollection declines with age [5], with older people less able to recall information successfully [6]. The association between intelligence and recall has not been shown to be significant, but a higher level of medical knowledge was associated with better recollection [5,8]. Memory and anxiety levels are connected to recollection in a Yerkes-Dodson curve (an inverse U curve): there is significantly better recollection at a moderate level of anxiety than at low or high levels of anxiety [5,7,8]. There also seems to be a linear association between the amount of information given and how much is recalled; the more information is provided, the more is lost [8].

The effectiveness of different ways of introducing information (i.e. written, oral, pictorial) has also been studied. Some studies have shown that the use of pictorial instructions is beneficial, although this has not been supported by others [5,6,9]. The combination of oral and written advice appears to be the most effective [6,7].

The type of information provided to the patient also affects recollection. Information related to diagnosis is remembered best, whereas the worst recall is of GPs' instructions and advice [5,8]. Patients tend to remember information better either if it is perceived as more important or if it is provided at the beginning of the consultation [5-8]. However, Watson and McKinstry, in their systematic review of intervention trials, did not identify any research that has explored the effect on recall of restricting the amount of information given at any one time, or of prioritizing important advice by placing it last in discussions, although there are good psychological reasons for believing these techniques may be effective [6].

Recollection is also related to the complexity of language used. Information given in simple language is supposedly better remembered [6,7,9,10]. These findings have implications for improving communication between doctors and their patients, and possibly for increasing the degree to which patients comply with medical recommendations [11]. Providing only general information with no specificities was associated with a decline in recall of 22-38% [5], while specific or detailed advice, rather than general, significantly improved recollection, the amount of remembered information increasing to 25-50% [5,6,12,13].

Because of the trends of increasing workload in general practice [1], time has also been studied. One of the potential consequences of increased workload is shortened consultation time [14-16]. This might lead to less detailed information provided by GPs, without repetition, which has an impact on both GPs' work and on patient satisfaction and understanding. The time factor may be very important in GP-patient communication, but it has not yet been fully explored. Consultation time is dependent on many factors: the physicians themselves [17], the patients, the reasons for the visit, the relationship between the physician and the patient, organizational and other.

Consultation time in general practice in Slovenia is very short; less than the mean consultation time in six European countries by almost 30% [18]. Due to this short consultation time, i.e. 7.08 ± 6.28 minutes [19], it would be reasonable to expect poorer GP-patient communication, which is less focused on specificities. The determinants of consultation time in Slovenia were studied by Petek Ster et al [19]. Longer consultation time was dependent on the characteristics of patients (female gender, higher age, higher level of education, change of GP within the last year, number of health problems); physicians and the organization of their work (higher age, absence of high workload); and type of visit (all visits with consultation and/or clinical examination).

Apparently GPs spend more time consulting women and the elderly [18,20-23]. However, only a small proportion of variability can be explained by the patient's age [20], which means that other factors, such as the number of health problems [18], the characteristics of the health problems, and the ''physician's speed'' are more important predictors of consultation time [23]. Consultation length is determined by variables related to the doctor and the doctor's country as well as by those related to patients. Women consulting in an urban practice with problems perceived as psychosocial have longer consultations than other patients [23]. Since more knowledgeable people recall more of the given advice [5,8], it is reasonable to expect that less well-educated people are at risk of not getting the necessary attention during their consultation, and are advised in a less appropriate manner.

Although psychological research which highlights techniques and factors postulated to influence recall exists [6], only a limited body of work has been conducted in a clinical context. Research focusing on the influence of gender on recall ability has been somewhat limited, while age has been shown to affect poorer recall [6]. Verbally communicating medical information with patients has the benefit of being quick, but written material should lead to greater recall [6]. Since much of the research carried out on memory has not been not conducted in a clinical setting [6], the aim of this study was to explore the factors related to the recall of GP advice after a short and a long delay between consultation and recall assessment, in "real life" conditions with limited resources. We were therefore unable to either video or audio record the consultations, nor ensure consistent timekeeping, which presents serious limitation to the study. However, we wanted to conduct the first study on recall in Slovenian family medicine, which might be considered as a preparation for more comprehensive research, covering patients with multi-morbidity in a representative sample of practices; and to identify the factors which determine how much of the GP's advice patients are able to recall, i.e. age, gender, education level, number of given instructions and time period.

## Methods

A prospective survey was conducted in an urban general practice in Slovenia.

The research protocol was approved by the Commission of the Republic of Slovenia for Medical Ethics, decision number 64/11/06, on November 4, 2006.

### Patients

Data were collected from January 1 2007 to June 1 2009. A random sample of general practice attendees, aged 18 years and above, who had visited their GP for health problems and were given a physical examination, were included in the study. Other inclusion criteria allowed only patients without dementia or even mild cognitive impairment, and who had only one complaint (health problem). The inclusion criteria were set in accordance with the results of representative data on Slovenian GPs' performance [19], which reported that the majority (65.9%) of patients are subjected to a physical examination and have an average of 1.57 ± 0.87 health problems per consultation. Since the study was constructed as a pilot research in the field of family medicine in Slovenia, and due to limited resources, only single issue health problems were dealt with, which seemed more feasible for a first step. Visits for administrative purposes (Petek Ster et al [19] reported 30.5% of these) were also excluded. Participation in the study was voluntary. Of 285 patients asked, 250 (87.7% response rate) agreed to participate.

### Procedure

In the *sampling period *(the first six months), a nurse measured consultation time, and asked every fifth patient who met the inclusion criteria if they were willing to repeat their given advice after leaving the consultation with the GP. She explained that a study on procedures in family care was being conducted and invited them to participate. The exact objective was not explained, and none of the sampled patients asked for further explanation; they were free to collaborate with the nurse in filling in the questionnaire, and were not influenced in any way. The data sheet was completed prior to leaving the practice.

The GP was not instructed to use any of the following recall-promoting physician behaviours (RPBs) [24]: repetition, categorization, summarization, technical term avoidance, importance emphasis, written materials, requested patient note taking, providing a rationale; the possible use of these RPBs was not controlled and analysed since the consultations were not audio-video recorded. The number and the content of given instructions was a result of the nature and complexity of the health problem in question, and was not arbitrarily limited or defined. All the advice given was oral only. However, at the end of each visit the GP was instructed to ask the same question: "Do you have any questions about what I have suggested?" or "Was everything I said clear to you?" After each consultation, the short-term recall of the medical instructions was tested by the nurse, who asked each patient to verbally recall the treatment instructions (a requested restatement) given by the GP. This short-term recall test took place within 1 minute after the consultation.

During the *follow-up period*, data collection (long-term recall testing) continued with those patients for whom the nurse had completed a summary sheet and whose immediate recall had already been tested. All 250 patients from the sampling phase were willing to participate in the follow-up. Follow-up visits to the practice were set at various times, depending on the nature of the problem presented at the first visit. The long-term recall of the medical instructions given at the previous visit was again tested by the nurse, who asked each patient to verbally recall the previous treatment instructions given by the GP.

### Materials

The summary sheet was designed to determine which advice was provided and recalled twice, once immediately after the examination (short-time recall) and again at the re-test on the second visit to the clinic (long-term recall). The date of the visit, the gender, age and education of the patient, the consultation time and the advice provided by the GP were recorded. The summary sheets were filled in by the nurse and were stored in a special folder, separate from the patients' medical records. In this way the GP was not informed which patients were participating in the survey, and so did not pay them any special attention when communicating with them. The GP's records followed the usual routine pattern. As the GP did not know which patients were participating, the nurse was responsible for completing the summary sheets and keeping them in order.

### Data analysis

In the data analysis, the patients' answers were compared with the GP's notes, transcribed from the medical records into the summary sheet by a nurse. Since simple communication defining diagnoses, prognoses and treatment advice was used, concrete and specific terms in the GP's notes and in the patients' answers were regarded as a correct recall of information, e.g. "take a rest for five days" ("rest", "five days"), with exact specification of the terms, e.g. "take xx prescription drug every six hours for the next seven days" ("xx prescription drug", "every six hours", "seven days"). Only the accuracy of the recalled instructions was tested. Patients' answers were assessed as to the amount of recalled information (one to four items), compared with the given information in the GP's notes. Answers were marked as "no recall" if patients recalled the information incorrectly or not at all.

When data collection was completed, all the fully compiled summary sheets and the GP's notes (medical records) were inspected by a senior GP to test the veracity of assessment. The comparison between the recorded patients' answers and the GP's notes was repeated. No summary sheets were eliminated due to discrepancies in the assessed recall.

The sample data were presented by frequencies and percentages for categorical variables, or by mean values and standard deviations (M ± SD) for continuous variables. The risk factors which lead to patients forgetting the GPs' instructions were calculated in multivariate binary logistic regression analysis. The calculation included the chi-square (χ^2^), odds ratio (OR), 95% confidence interval (95% CI) and P value. Statistical analysis was performed with the SPSS 15.0 software. P < 0.05 was marked as statistically significant.

## Results

### The sample

The sample consisted of 250 patients aged over 18 years. Most of them were women (174 (69.6%), while about one third were men (76 (30.4%)). The mean age of the patients was 58.1 ± 11.3 years, the youngest being 26 and the oldest 83 years old. The majority of patients had completed high school education (148 (59.2%)) with 69 (27.6%) patients having college or a university education, while 33 (13.2%) had only primary school (which ends at age 14 in Slovenia).

### The sampling period

All patients received at least one and up to four instructions (2.4 ± 0.8), divided in the following way: one instruction (7.6%); two instructions (52.0%); three instructions (32.0%); four instructions (8.4%). Immediately after leaving the GP's office, short-term recall was tested by the nurse, who requested a verbal restatement of the medical advice from each patient who was willing to participate in the survey. The results showed that at the first visit, when we studied short-term recall, the number of given instructions did not affect how many were recalled. Patients were given a maximum of four instructions and were, as expected, able to recall all of them immediately, a short-time recall of 100%. The consultation time was 6.47 ± 3.92 minutes. However, due to missing data in the recording of consultation time, it was not included in the further modelling process.

The number of days between the first and second visits to the clinic (the re-test interval) was calculated at 47.4 ± 35.2 days, which suggests that the observed parameter values were very scattered. The re-test intervals ranged between 1 and 178 days.

### The re-test period: Multivariate binary logistic regression modelling

In the re-test a group of 80 (32.0%) forgot some instructions; most patients forgot one instruction (71 (28.4%)), and a small number forgot a maximum of two (9 (3.6%)). Multivariate binary logistic regression modelling was used to identify the determinants of the quantity of recalled instructions at the re-test. The logistic regression model is shown in Table 1. The greatest risk factor for patients forgetting instructions was if they were given three (OR 26.1, 95% CI 3.15-215.24, p = 0.002), or four instructions (OR 56.8, 95% CI 5.91-546.12, p < 0.001). An association was demonstrated between the amount of recalled information and education level; high school (OR 0.4, 95% CI 0.15-0.99, p = 0.049) and college education (OR 0.3, 95% CI 0.10-1.00, p = 0.050) reduced the risk of patients forgetting given instructions, while a longer re-test interval (15-30 days, 31-60 days and more than 60 days) increased the likelihood (OR 3.3, 95% CI 1.06-10.13, p = 0.040; OR 3.2, 95% CI 1.28-8.07, p = 0.013; OR 2.5, 95% CI 1.05-6.02, p = 0.038, respectively).

**Table 1:** Determinants of Long-term Recall: Logistic Regression Model of the Association between the Number of Instructions Recalled (1 to 4), Patients' Characteristics and the Re-Test Interval (χ^2 ^= 65

Characteristic	All n = 250 (%)	Instructions recalled		OR with 95% CI	p-value
		Yes n = 170 (%)	No n = 80 (%)		
**Gender**					
male	76 (30.4)	53 (31.2)	23 (28.8)	1.00	
female	174 (69.6)	117 (68.8)	57 (71.3)	0.93 (0.45-1.91)	0.835
**Age (years)**					
50 or less	69 (27.6)	51 (30.0)	18 (22.5)	1.00	
51-64	99 (39.6)	68 (40.0)	31 (38.8)	1.28 (0.61-3.12)	0.445
65 and above	82 (32.8)	51 (30.0)	31 (38.8)	1.32 (0.54-3.22)	0.537
**Education**					
primary school	33 (13.2)	18 (10.6)	15 (18.8)	1.00	
high school	148 (59.2)	102 (60.0)	46 (57.5)	0.39 (0.15-0.99)	0.049
college	69 (27.6)	50 (29.4)	19 (23.8)	0.32 (0.10-1.00)	0.050
**Number of given instructions**					
1	19 (7.6)	18 (10.6)	1 (1.3)	1.00	
2	130 (52.0)	108 (63.5)	22 (27.5)	4.02 (0.49-32.62)	0.193
3	80 (32.0)	38 (22.4)	42 (52.5)	26.05 (3.15-215.24)	0.002
4	21 (8.4)	6 (3.5)	15 (18.8)	56.80 (5.91-546.12)	< 0.001
**Re-test interval (days)**					
14 or less	59 (23.6)	47 (27.6)	12 (15.0)	1.00	
15-30	35 (14.0)	23 (13.5)	12 (15.0)	3.27 (1.06-10.13)	0.040
31-60	68 (27.2)	42 (24.7)	26 (32.5)	3.21 (1.28-8.07)	0.013
more than 60	88 (35.2)	58 (34.1)	30 (37.5)	2.52 (1.05-6.02)	0.038

Nagelkerke R^2 ^= 0.323

OR: odds ratio

CI: confidence interval

The multivariate logistic regression model (χ^2 ^= 65.667, df = 11, p < 0.001) explained 32% of the variance of the amount of GPs' instructions recalled by patients at the re-test, when given between one and four instructions (Nagelkerke R^2 ^= 0,323; p < 0,001), with 80.6% sensitivity and 65.2% specificity.

## Discussion

### Statement of principal findings

This study addressed the influence of the age, gender, and education level of patients; the consultation time; the number of given instructions; and the time between the first and second visit, on the amount of medical advice recalled (long-term recall) in patients with a single issue health problem. The determinants of better recall were high school and college education, while poorer recall was determined by the number of given instructions (three or four) and a re-test interval of more than 14 days.

### Strengths and weaknesses of the study

It was possible to explain only 32% of the variance, with multivariate binary logistic regression modelling used to identify the determinants of the number of recalled instructions at the re-test. The authors are well aware that other factors (e.g. the form of the instruction, the type of information, and the level of anxiety in patients) are known to influence recall [6], but their inclusion would require a more complex research design and were therefore not studied on this occasion. Due to limited resources it was only possible to conduct a study strictly reflecting the practical work of a GP in Slovenia, without additional technical equipment (i.e. audio-video monitoring), training and/or personnel.

The GP was not aware which patients were included in the survey so that the research goals would not be compromised, and therefore did not give any of them any special attention. As they were not informed of the aim of the survey, the patients were not particularly focused on memorizing the GP's advice. Apart from the nurse's work with the summary sheets and medical records, "real-life" family clinic procedures were maintained. During the consultations, neither the GP's nor the patient's attention was affected by the data collection; in this way biased results were avoided.

The strength of the study is therefore in the naivety of the patients, who had not been instructed to focus on the long-term recall of medical instructions. Given that, we were able to re-test the amount of recalled information in a natural environment, without pressuring the patients to achieve better results at the second (re-test) consultation.

Our field study was conducted at a family clinic without any additional load on top of the GP's routine work. Since the design of the study was not experimental, it has both limitations and advantages. The survey was conducted at one family practice and therefore the findings may not be representative for the whole of Slovenia. However, the model illuminates the relationship between the factors affecting long-term recall. The results confirmed the way many GPs intuitively behave: the doctors should give a little advice; if more is needed, other ways to educate the patients should be implemented (e.g., healthy lifestyle programmes, workshops for coping with asthma or diabetes).

### Strengths and weaknesses in relation to other studies

In their recent study, McKinstry et al [25] sought interactions between accurate recall, consultation type and factors postulated to influence recall, and reported poorer recall in patients presenting multiple problems, with brain injury or a low memory score. Since our study excluded patients with multiple problems, and the findings reflect single health problem situations, recalling information when dealing with more health complaints in patients is still to be studied. When four instructions were given to the patients, the GP could be almost certain that patient would not recall one or two of them (Table 1); on the other hand, patients might be advised to restrict the number of problems they present in any one consultation [25].

Although precise recall of medical instructions is not sufficient basis for engagement and participation in the healing process, it is very much required. Patients who are unable to recall at least part of the GP's instructions will most probably not be able to use them in the appropriate situation. Assuming that 40-50% of patients inadequately carry out their doctor's advice [13] since they do not recall it, memorizing medical information is a prerequisite for proper implementation of the doctor's instructions. Between 40-80% of information given by medical staff is reported to be immediately forgotten [5,26], although was not proven in our study, which demonstrated 100% immediate (short-term) recall. This is probably at least partly due to the limited amount of information given by the doctor. We should also bear in mind that additional interviews (i.e. the short-term recall test in our study) have been shown to result in patients forgetting less and remembering more accurately [6], which might have affected their long-tem recall. However, almost half of the information recalled is incorrect [5,26], which might be explained by the GPs' behaviour (e.g. the use of medical terminology difficult to understand), the form of the information provided (spoken or written), or the patients' specific expectations, e.g. an explanation of the problem [7]. It has been already reported by Zebiene et al [27] that GPs should put more emphasis on identifying and addressing patient expectations of understanding and explanation, and of emotional support in primary care consultations, in order to improve the overall quality of care. Meeting patients' expectations of getting more thorough explanations means providing more specificities or details which have been shown to significantly improve recollection [5,6,13].

We expected that the age of the patients would play an important role in the recollection process, but the results showed otherwise (Table 1). This finding is concordant with Jansen et al [26], who found that immediately after the consultation, the younger patients correctly recalled more information, but during the re-test period the difference in recalled information compared to older patients had disappeared. In general, this could be explained by the incidence of multi-morbidity in older people, which may affect memory (e.g. fatigue, depression). The elderly may have problems processing new data, and a lower capacity of short term memory; while in our survey it might also be due to inclusion criteria, i.e. only one health complaint. Although several authors have stated that since elderly have already experienced many life situations they are therefore able to compensate for these deficits by experience [5,7,10,28] we embrace the conclusion that older people are less able to recall information successfully due to deficits in data encoding processes [6]. In relation to this, recall in older individuals may be assisted by the repetition of information and the provision of written material [13].

In comparison to a representative sample of Slovenian general practice attendees, described by Svab et al [29], the patients in our sample were older (58.1 ± 11.3 years *vs*. 51.7 ± 19.0), better educated (primary school 13% *vs*. 41%; high school 59% *vs*. 48%; college or university degree 28% *vs*. 11%), with a higher proportion of women (69.6% *vs*. 54.8%) and shorter consultation time (6.47 ± 3.92 minutes *vs*. 7.08 ± 6.28 minutes). However, consultation time in our study should be considered with great caution, while age was not shown to be associated with the amount of recall (Table 1). Aside from the amount of given advice, level of education was identified as a relevant predictor of recall in patients, although level of education alone does not reflect memory potential [5,8,9].

In our survey, the number of instructions provided was not defined in advance, as it was determined according to the needs of the patient during the consultation. The results showed that at the first visit, the number of given instructions did not affect how many were recalled. The association between the number of given instructions and the amount of recalled information was however seen at the follow-up consultation. Our findings on long-term recall are consistent with the reviewed literature: an association was found between the number of given instructions and the amount of recalled information after some time. Since the re-test interval used for the purpose of analysis was very variable, the connection might have been more pronounced if patients had been given more than four instructions or the re-test interval had been more homogeneous.

As stated by Ley [4], a reduction in the amount of forgotten information can be achieved by the use of simpler language, explicit categorization of information, repetition, and concrete-specific rather than general-abstract advice statements. Studies suggest that patients forget a great deal of important information and that recall can be increased through recall-promoting behaviours (RPBs) like repetition or summarization [24]. In our study design RPBs were not controlled and the GP was not trained to apply them. Since most RPBs were used inconsistently, several RPBs that are particularly helpful were not utilized; moreover, RPBs might have been used inefficiently, aside from an assessment of patient understanding (the GP's routine check of patient understanding) and a requested restatement during the completion of the summary sheet with the nurse. A proportion of the unexplained variance may be due to this fact.

### Meaning of the study: possible mechanisms and implications for clinicians or policymakers

Effective treatments can be rendered useless by poor patient recall of treatment instructions. Moreover, insufficient understanding and recall of instructions reduce patient satisfaction and commitment to treatment [4,5]. Therefore our finding that the greatest risk of patients forgetting GP's instructions was represented by three, or even worse, four instructions (Table 1), could be used as a recommendation for GPs.

### Unanswered questions and future research

The fact that the nurse asked the patients to repeat the recommendations probably affected their long-term recall. In further research, the use of immediate recall in patients should be controlled, as well as the association between the number of health problems, the number of recalled instructions and the effectiveness of the treatment. As emphasized by Silberman et al [24], when examining which RPBs are applied, consultation length should be taken into account. Since consultation time in Slovenia is very short [19], simple principles guiding RPBs' use are needed to help GPs apply these communication tools effectively.

We were able to show that the quantity of recalled information after an elapsed time period depends on the degree of patient education, which was unexpected, since the reviewed literature did not show this association. In Slovenia, the consultation time for higher-educated patients was shown to be longer than for less well-educated patients [19]; due to missing data we were not able to test the association between the consultation time, the education level of patients and their recall, therefore further study is needed. It might also be necessary to consider the patients' medical knowledge as a factor affecting the recollection of medical information and instructions [5,8].

The limited number of given instructions (up to four) was insufficient to show the bigger picture of the association between a larger number of instructions and poorer recollection. Since multi-issue health problems were not included, and only single issue complaints were taken into account, another variable to be studied thoroughly is the number of health problems in patients.

Bearing in mind that different medical conditions and situations require individual advice, the results of recall might have been influenced by the complexity of the underlying disease in patients, although the number of instructions was limited to four. This is another limitation to be overcome in further work. The impact of education, in association with systematically applied RPBs, and an assessment of treatment efficiency should also be studied in a more complex research design in the future. In that case, the proportion of explained variance, 32% in our survey, might be larger.

## Conclusions

The greatest risk of patients forgetting medical instructions was represented by three or four instructions. The amount of recalled information was better in higher educated patients. The study sample was relatively small and cannot be considered representative of Slovenian general practice. Nevertheless, the relative importance of the factors that influenced recall is independent of the representativeness of the sample, and may therefore be offered for GPs' guidance in their daily work: the study indicates that patients should be given only one, or maybe two, pieces of advice during a general practice consultation. Where more advice is needed, the next consultation should be scheduled in the short term (up to 14 days), and perhaps only to more well-educated patients. Since the level of education is marked on patients' medical charts, it would be possible for GPs to take this characteristic into consideration when providing either more complex or a larger volume of medical information.

For complex health problems encountered in general practice (e.g. newly diagnosed chronic disease), other methods may be more useful (e.g. patient education provided by nurses, special clinics etc.). Gender and age by themselves do not need to be considered; however empowerment in communication skills, with special emphasis on RPBs in GPs, is to be recommended.

## Competing interests

The authors declare that they have no competing interests.

## Authors' contributions

PS participated in the execution of the study, carried out the coordination, and drafted the manuscript. IS participated in the data interpretation and helped to draft the manuscript. MR conceived the study and participated in the data collection. NKG participated in the data evaluation. All the authors read and approved the final manuscript.

## Pre-publication history

The pre-publication history for this paper can be accessed here:

http://www.biomedcentral.com/1471-2296/12/141/prepub
